# Preoperative Altered Spontaneous Brain Activity and Functional Connectivity Were Independent Risk Factors for Delayed Neurocognitive Recovery in Older Adults Undergoing Noncardiac Surgery

**DOI:** 10.1155/2020/9796419

**Published:** 2020-06-16

**Authors:** Zhaoshun Jiang, Xixue Zhang, Yating Lv, Xiaodong Zheng, Huibiao Zhang, Xuelin Zhang, Chongyi Jiang, Guangwu Lin, Weidong Gu

**Affiliations:** ^1^Department of Anesthesiology, Huadong Hospital Affiliated to Fudan University, Shanghai 200040, China; ^2^Key Laboratory of Clinical Geriatric Medicine, Shanghai 200040, China; ^3^Institutes of Psychological Sciences, Hangzhou Normal University, Hangzhou, 311121 Zhejiang, China; ^4^Department of Radiology, Huadong Hospital Affiliated to Fudan University, Shanghai 200040, China; ^5^Department of Thoracic Surgery, Huadong Hospital Affiliated to Fudan University, Shanghai 200040, China; ^6^Department of General Surgery, Huadong Hospital Affiliated to Fudan University, Shanghai 200040, China

## Abstract

**Objectives:**

Recently, it has been demonstrated that patients with subtle preexisting cognitive impairment were susceptible to delayed neurocognitive recovery (DNR). This present study investigated whether preoperative alterations in gray matter volume, spontaneous activity, or functional connectivity (FC) were associated with DNR.

**Methods:**

This was a nested case-control study of older adults (≥60 years) undergoing noncardiac surgery. All patients received MRI scan at least 1 day prior to surgery. Cognitive function was assessed prior to surgery and at 7-14 days postsurgery. Preoperative gray matter volume, amplitude of low-frequency fluctuation (ALFF), and FC were compared between the DNR patients and non-DNR patients. The independent risk factors associated with DNR were identified using a multivariate logistic regression model.

**Results:**

Of the 74 patients who completed assessments, 16/74 (21.6%) had DNR following surgery. There were no differences in gray matter volume between the two groups. However, the DNR patients exhibited higher preoperative ALFF in the bilateral middle cingulate cortex (MCC) and left fusiform gyrus and lower preoperative FC between the bilateral MCC and left calcarine than the non-DNR patients. The multivariate logistic regression analysis showed that higher preoperative spontaneous activity in the bilateral MCC was independently associated with a higher risk of DNR (OR = 3.11, 95% CI, 1.30-7.45; *P* = 0.011). A longer education duration (OR = 0.57, 95% CI, 0.41-0.81; *P* = 0.001) and higher preoperative FC between the bilateral MCC and left calcarine (OR = 0.40, 95% CI, 0.18-0.92; *P* = 0.031) were independently correlated with a lower risk of DNR.

**Conclusions:**

Preoperative higher ALFF in the bilateral MCC and lower FC between the bilateral MCC and left calcarine were independently associated with the occurrence of DNR. The present fMRI study identified possible preoperative neuroimaging risk factors for DNR. This trial is registered with Chinese Clinical Trial Registry ChiCTR-DCD-15006096.

## 1. Introduction

Delayed neurocognitive recovery (DNR) is a common and well-known neurological complication following a procedure, especially in older adults [1, 2]. Although primarily described after cardiac surgery, DNR has also been reported following major noncardiac surgery [3, 4]. The occurrence of DNR following a procedure is associated with multiple adverse outcomes, including poor functional recovery and prolonged hospitalization [2].

Recently, it was suggested that patients with subtle preexisting cognitive impairment were more susceptible to postoperative cognitive impairment [5, 6]. These findings implied that preoperative cognitive function decline is an important risk factor for neurocognitive disorders following surgery and anesthesia [7]. Numerous studies have demonstrated that cognitive function was closely related to individual brain structure and function [8]. Advancements in magnetic resonance imaging (MRI) have provided a useful tool to noninvasively capture detailed alterations in brain structure and function [9]. In our pilot study with small sample size, we found that there were differences of regional homogeneity in the right hippocampus/parahippocampus between the DNR patients and non-DNR patients (Gaussian random field corrected) [10].

Voxel-based morphometry is used to detect subtle change in gray matter with voxel-by-voxel comparisons [11]. Studies using voxel-based morphometry analysis have demonstrated that changes in gray matter volume were associated with cognitive decline [11, 12]. However, the key regions with preoperative changes in the brain structure in DNR patients have not been fully elucidated and findings from previous studies need to be further validated. Resting-state functional MRI (rs-fMRI) has emerged as a promising approach to explore changes in brain function. Amplitude of low-frequency fluctuation (ALFF, 0.01-0.08 Hz), which detects the amplitude of blood oxygen level-dependent signal relative to the baseline, can be used to measure the intensity of spontaneous fluctuations in the brain [13]. ALFF analysis has been widely used to investigate the neural underpinnings of various diseases associated with cognitive dysfunction, including mild cognitive impairment, Alzheimer disease, and attention-deficient hyperactivity disorder [13–15]. Functional connectivity (FC), which reflects synchronous spontaneous fluctuations between brain regions, can provide information about functional integrity of brain networks [16]. The FC analysis is a useful tool to investigate cognitive dysfunction disorders [17, 18]. The spontaneous fluctuations reflect localized neural activity, whereas FC provides information on correlated activity between two brain regions. The two methods are complementary to each other and provide a full perspective of the brain responses during rest [19]. Therefore, these approaches may help elucidate the characteristics of preoperative resting-state brain activity in DNR patients.

In the present study, we hypothesized that there were preoperative abnormalities in the brain structure and brain function in older patients who developed DNR after noncardiac surgery. We investigated the differences in preoperative gray matter volume, ALFF, and FC between the patients with and without DNR. The association between neuroimaging results and the occurrence of DNR was also analyzed to identify preoperative neuroimaging risk factors for DNR.

## 2. Materials and Methods

### 2.1. Study Design and Ethics

This was a nested case-control study approved by the Ethics Committee of Huadong Hospital Affiliated to Fudan University with the approval number of 20170020. This study was registered at Chinese Clinical Trial Registry (http://www.chictr.org.cn/) with the identifier of ChiCTR-DCD-15006096 on 16th March 2015.

All patients were recruited 1-3 days prior to surgery. Written informed consent was obtained from patients or their legal representatives prior to surgery. All patients received an MRI scan at least 1 day before surgery. Cognitive function was assessed prior to and 7-14 days following surgery using a battery of neuropsychological tests [2]. DNR was diagnosed according to the method recommended by the International study of postoperative cognitive dysfunction (ISPOCD1) definition [4]. Subsequently, patients were divided into the DNR group and non-DNR group. The demographics, voxel-based morphometry data, ALFF data, FC data, and neuropsychological tests results were assessed and analyzed.

### 2.2. Participants

Patients were recruited from September 2017 to February 2019 at the Huadong Hospital Affiliated to Fudan University. Patients were included if they met all the following criteria: (1) patients scheduled to undergo noncardiac surgery with an expected duration of more than 2 hours, under general anesthesia; (2) age ≥ 60 years; (3) American Society of Anesthesiologists classification I-III; and (4) right handedness.

Patients were excluded if they met any of the following criteria: (1) education duration < 6 years; (2) Mini-Mental State Examination (MMSE) score prior to surgery < 24 points; (3) preexisting mental and/or psychiatric disease; (4) Parkinson's disease; (5) history of cardiac and/or central nervous system vascular disease; (6) history of cardiac and cranial surgeries; (7) taking sedatives or antidepressants during the nearest year; (8) alcohol or drug abuse; (9) severe hepatic or renal dysfunction; (10) vision and audition impairment or language troubles impeding communication; (11) situations unsuitable for an MRI scan; and (12) unwillingness to complete repeat neuropsychological tests.

### 2.3. Anesthesia Protocols

Anesthesia was induced using propofol, sufentanil, and rocuronium through a central intravenous catheter. Anesthesia was maintained with propofol and remifentanil. Intravenous rocuronium and sufentanil were used when necessary.

Bispectral Index Score was maintained at 40 to 60 to ensure appropriate anesthesia depth. Heart rate, arterial pressure, SpO_2_, PETCO_2_, and body temperature were monitored continuously. Intravenous patient-controlled analgesia was used to keep the visual analogue scale score < 3 after surgery, and the patient-controlled analgesia formula contained sufentanil and ketorolac tromethamine.

### 2.4. Neurocognitive Assessment

All patients were assessed at least 1 day prior to surgery and again at 7-14 days following surgery when patients returned to the hospital for suture removal. Testing was performed by a trained researcher in a quiet environment. The neuropsychological test battery consisted of 6 tests: MMSE (orientation, memory, attention, and visuospatial ability), Digit Span Forwards (DSF, short-term memory and attention), Digit Span Backwards (DSB, working memory and attention), Digit Symbol Substitution Test (DSST, working memory, executive function, visual-motor coordination, visual-spatial scanning and attention), Verbal Fluency Test (VFT, verbal memory and associative ability), and Trail Making Test-part A (TMT-A, visual search speed abilities, attention and executive function).

A *Z*-score was calculated for each single test according to the method recommended by the ISPOCD1 definition to delineate patients' postoperative alterations in cognitive function [4]. To correct for the learning effect of repeatedly tests, 30 healthy volunteers with age and education duration matched to the patient group were enrolled. The inclusion and exclusion criteria were the same as those for patients, except for the anesthesia and surgery procedure. The volunteers underwent neuropsychological tests at the same time intervals corresponding to assessments in the patients undergoing surgery. We compared changes for each test from baseline scores to follow-up scores in volunteer subjects to obtain the average learning effects (*Δ*xc) and their standard deviations [SD(*Δ*xc)]. For patients, we calculated the changes from baseline scores to follow-up scores. The changes (Δ*x*) subtracted the average learning effects, and divided the results by the volunteer subjects' SD to obtain a *Z* score for each test. The *Z* score could be built as follows:

(1) $$\begin{array}{c} Z=\frac{\Delta x-\Delta \text{xc}}{\text{SD}\left(\Delta \text{xc}\right)}. \end{array}$$

The *Z* scores of all tests in an individual patient were then summarized and divided by the SD for this sum of *Z* scores in volunteer subjects, creating a composite *Z* score. The composite *Z* score could be built as follows:

(2) $$\begin{array}{c} \text{composite }Z\text{ score}=\frac{\sum Z}{\text{SD}\left(\sum Z_{\text{c}}\right)}. \end{array}$$

Large positive *Z* scores or composite *Z* score represented deterioration in cognitive function from baseline. DNR was defined as follows: at least two of the neuropsychological test *Z* scores were >1.96 or the composite *Z* score was >1.96.

### 2.5. MRI Data Acquisition

All patients received an MRI scan at least 1 day prior to surgery on a 3.0 T MRI scanner (SIEMENS Skyra). Participants were instructed to relax with their eyes closed, stay awake, and remain motionless during the MRI data acquisition. For each patient, the MRI scanning protocol included following sessions: (1) 3D high-resolution T1-weighted anatomical images were acquired using a 3D-MPRAGE sequence: 176 sagittal slices, TR = 1900 ms, TE = 3.57 ms, voxel size = 1 mm × 1 mm × 1 mm, and flip angle = 9°; and (2) rs-fMRI data was acquired using an echo-planar imaging sequence: 33 axial slices, slices of thickness = 4 mm with 0 mm gap, TR = 3,000 ms, TE = 30 ms, voxel size = 3.4 mm × 3.4 mm × 4 mm, and flip angle = 90°. In this scan, 120 volumes were obtained.

### 2.6. Voxel-Based Morphometry Processing and Analysis

Data was analyzed with the VBM8 toolbox in Statistical Parametric Mapping version 8 (SPM8, The Wellcome Trust Centre for Human Neuroimaging, London, UK) in MATLAB version R2013b (MathWorks, Inc., Natick, MA). The images were segmented into gray matter, white matter, and cerebrospinal fluid (CSF). After being modulated and normalized, images were smoothed with an 8 mm × 8 mm × 8 mm full-width half-maximum (FWHM) Gaussian kernel. The smoothed gray matter images were used to perform the voxel-by-voxel comparison of gray matter volume between the DNR patients and non-DNR patients.

### 2.7. rs-fMRI Data Preprocessing and ALFF Analysis

Functional imaging data were processed using SPM8 and RESTplus version 1.22 (Institutes of Psychological Sciences, Hangzhou Normal University, China) in MATLAB R2013b [20]. Each participant's first 5 volumes were discarded to make the longitudinal magnetization reach a steady state and to avoid potential noise related to the participant's adaptation to the scanner. The remaining images were slice-time corrected and realigned for head motion correction. The corrected images were further spatially normalized to the Montreal Neurological Institute (MNI) 152 template (resampling voxel size = 3 mm × 3 mm × 3 mm) and spatially smoothed with a 6 mm × 6 mm × 6 mm FWHM Gaussian kernel. After the linear trend of time course removal, the imaging data were then temporally band-pass filtered (0.01–0.08 Hz) to remove the effects of low-frequency drift and high-frequency noise. The motion artifact, white matter signal, and CSF signal were regressed out as nuisance covariates.

ALFF analysis was performed using RESTplus 1.22. The filtered time series was converted to the frequency domain using the fast Fourier transformation analysis, and the power spectrum was then obtained [13]. The square root of the power spectrum was then calculated and averaged across 0.01–0.08 Hz for each voxel. This averaged square root was taken as the ALFF. For standardization purposes, ALFF of each voxel was divided by global mean ALFF values.

### 2.8. FC Analysis of ALFF-Based Seeds

The preprocessing of the FC analysis protocol was the same as in the ALFF analysis, except that the images were treated with the global mean signal for additional nuisance covariates. This study found abnormal ALFF values in the bilateral middle cingulate cortex (MCC) and left fusiform gyrus (FFG). Thus, the bilateral MCC and left FFG clusters were used as the regions of interest (ROI). The MNI coordinates of the center of the spherical ROI (radius = 6 mm) were determined by the peak *T*-value detected at the bilateral MCC and left FFG. For FC analysis, the mean time series was extracted from the seed region and correlated with the time series of each voxel of the whole brain for each subject. Individual *r*-maps were normalized to *Z* maps by using Fisher's *Z* transformation.

### 2.9. Statistical Analysis

Continuous variables were analyzed using the two-sided Student *t*-test or Mann-Whitney test. Categorical variables were analyzed using the *χ*^2^ test, continuity correction *χ*^2^ test, or Fisher's exact test. A *P* value < 0.05 was considered statistically significant. Statistical analyses were performed using SPSS version 22.0 (IBM, Armonk, NY, USA).

We compared the differences in neuropsychological test scores at 7-14 days' follow-up between the two groups, with baseline scores as a covariate. Group comparisons of preoperative gray matter volume, ALFF, and FC between the DNR group and non-DNR group were performed by using a two-sample Student *t*-test, with age, sex, educational duration, and preoperative cognitive function as covariates. The results were corrected using cluster-based false discovery rate (FDR) [21] with uncorrected voxel *P* < 0.001 and corrected cluster *P* < 0.05.

Partial correlation analysis was used to assess the correlations between MRI data and baseline cognitive data after adjusting for age, sex, and education duration. The correlations were considered significant at a threshold of *P* < 0.05.

Odds ratios (OR) and its 95% confidence intervals (CI) were calculated by logistic regression analysis to identify the independent risk factors associated with DNR. Candidate variables with a *P* < 0.15 on the univariate analysis were incorporated in the final multivariable model. Age was forced into the multivariate model, because age was considered clinically relevant to the occurrence of DNR.

## 3. Results

### 3.1. Subject Characteristics

**Figure 1:**
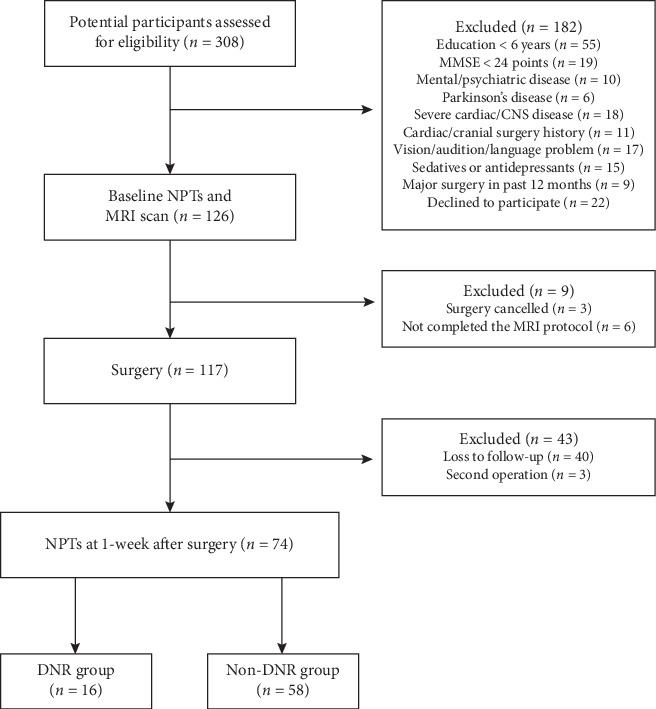
Strengthening the Reporting of Observational Studies in Epidemiology (STROBE) diagram. CNS: central nervous system; DNR: delayed neurocognitive recovery; MMSE: Mini-Mental State Examination; MRI: magnetic resonance imaging; NPTs: neuropsychological tests.

From September 2017 to February 2019, 126 patients aged 60 and older undergoing noncardiac surgery were enrolled in this study. Finally, 74 patients completed the MRI scan and the whole battery of neuropsychological tests pre- and postoperatively. The flow chart of patients through the study and detailed reasons for exclusion are provided in Figure 1.

DNR was identified in 16 of the 74 patients (21.6%). The patients with DNR had significantly shorter education duration than the patients without DNR (*P* = 0.002). There were no differences in age, sex, height, weight, body mass index (BMI), smoking status, surgery history, comorbidities, surgical duration, nature of surgery, and dose of intraoperative anesthetics between the DNR group and non-DNR group (Table 1).

### 3.2. Cognitive Function

There were no significant differences in neuropsychological test baseline scores between the two groups, except for DSST baseline score (*P* = 0.038). However, there were significant differences in follow-up scores of MMSE, DSF, DSB, DSST, VFT, and TMT-A between the DNR group and non-DNR group (*P* < 0.05) (Table 2).

### 3.3. MRI Findings of the Brain

**Figure 2:**
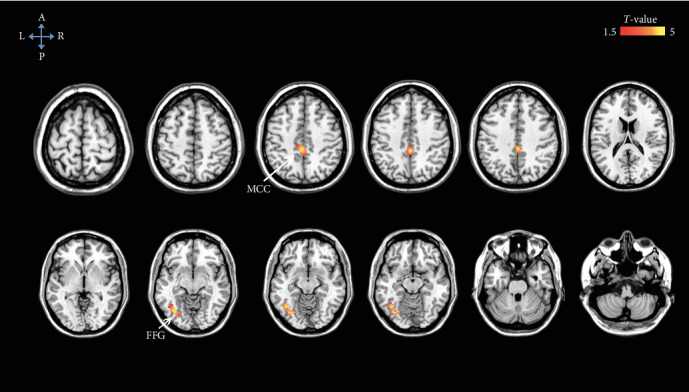
Brain regions exhibiting higher preoperative ALFF values in the DNR patients compared to those in the non-DNR patients, after adjusting for age, sex, education duration, and preoperative cognitive function. The statistical threshold was set at an uncorrected voxel *P* < 0.001 and a corrected cluster *P* < 0.05 (cluster-based FDR corrected). ALFF: amplitude of low-frequency fluctuations; DNR: delayed neurocognitive recovery; FDR: false discovery rate; FFG: fusiform gyrus; MCC: middle cingulate cortex.

**Figure 3:**
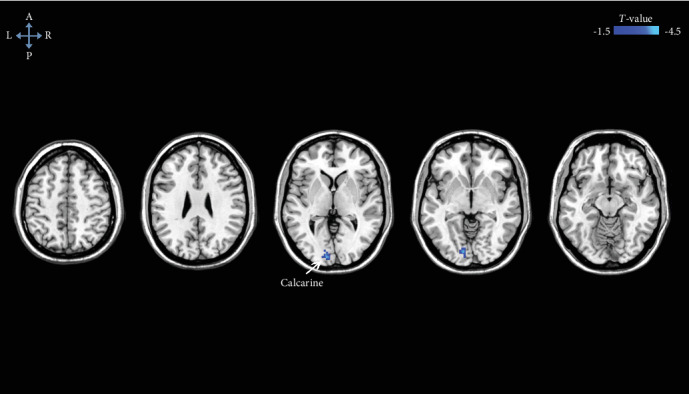
Brain regions exhibiting lower preoperative FC to bilateral MCC in the DNR patients compared to those in the non-DNR patients, after adjusting for age, sex, education duration, and preoperative cognitive function. The statistical threshold was set at an uncorrected voxel *P* < 0.001 and a corrected cluster *P* < 0.05 (cluster-based FDR corrected). DNR: delayed neurocognitive recovery; FC: functional connectivity; FDR: false discovery rate; MCC: middle cingulate cortex.

There were no brain areas showing significant differences in gray matter volume between the DNR patients and non-DNR patients. However, the DNR patients exhibited higher preoperative ALFF signals, with the peak differences in the bilateral middle cingulate cortex (MCC) and left fusiform gyrus (FFG) than the non-DNR patients (Figure 2 and Supplemental Table 1). Additionally, we found a significantly lower preoperative FC between the bilateral MCC and left calcarine in the DNR patients compared to that in the non-DNR patients (Figure 3 and Supplemental Table 1). Age, sex, education duration, and preoperative cognitive function were included as covariates in the voxel-based morphometry, ALFF, and FC analysis. The statistical threshold was set at an uncorrected voxel *P* < 0.001 and a corrected cluster *P* < 0.05 (cluster-based FDR corrected).

### 3.4. Correlation Analysis

After adjusting for age, sex, and education duration, we did not find any correlations between baseline cognitive scores and ALFF values in the bilateral MCC or ALFF values in the left FFG. In addition, there were no significant correlations between baseline cognitive scores and the MCC-calcarine FC values (*P* > 0.05, partial correlation) (Supplemental Table 2). The results indicated that preoperative spontaneous activity or FC was not related to baseline cognitive function.

### 3.5. Risk Factors for DNR

After the univariate analyses, five variables including education duration, sex, ALFF values in the bilateral MCC and left FFG, and FC values between the bilateral MCC and left calcarine were included into the subsequent multivariate logistic regression.

According to the multivariate logistic regression analysis, three variables were identified as independent variables correlating with DNR at 7-14 days following surgery (Table 3). Higher preoperative spontaneous activity in the bilateral MCC was independently associated with a higher risk of DNR (OR = 3.11, 95% CI, 1.30-7.45; *P* = 0.011). A longer education duration (OR = 0.57, 95% CI, 0.41-0.81; *P* = 0.001) and higher preoperative FC between the bilateral MCC and left calcarine (OR = 0.40, 95% CI, 0.18-0.92; *P* = 0.031) were independently correlated with a lower risk of DNR.

## 4. Discussion

The present study demonstrated that preoperative abnormalities in the brain function existed in patients who went on to develop DNR. The key findings included the following: (1) the DNR patients exhibited higher preoperative ALFF signals in the bilateral MCC and left FFG and lower FC between the bilateral MCC and left calcarine than the non-DNR patients, after adjusting for age, sex, education duration, and preoperative cognitive function; (2) there were no brain areas showing significant differences in gray matter volume between the DNR patients and non-DNR patients; and (3) the multivariate logistic regression analysis showed that preoperative higher ALFF in the bilateral MCC and lower FC between the bilateral MCC and left calcarine were independently associated with the occurrence of DNR. Taken together, the results suggested that older patients with altered preoperative brain function were more susceptible to deterioration in cognitive function following major noncardiac surgery.

Recently, molecular biomarkers in serum or CSF were shown to be predictive of susceptibility to postoperative cognitive decline. It was found that patients with postoperative cognitive dysfunction had increased preoperative serum levels of neuron-specific enolase [22], which is an indicator of neuronal injury. In addition, increasing evidence has supported that the preoperative CSF A*β*/tau ratio was associated with postoperative cognitive changes, suggesting that CSF biomarkers reflecting neuronal damage may exist prior to the appearance of significant cognitive deficits [23, 24]. The present study provided new evidence that premorbid changes in neuropathology exist in patients who developed DNR.

In the present study, we found that higher levels of preoperative spontaneous activity in the bilateral MCC were independently associated with a higher risk of DNR. The MCC is an important component of the limbic system that plays the role of a bridge between higher and lower levels of cognitive processing [25]. A large body of evidence supports the notion that the MCC is a heterogeneous area involved in multimodal cognitive functions such as executive function, working memory, attention, novelty detection, and feedback-mediated decision-making [26–29]. A study examining working memory reported that the MCC was activated by performing working memory tasks [30]. Moreover, it has been reported that the MCC was activated during divided attention or shifting between tasks, while MCC dysfunction has been shown to play a primary role in producing inattention and contribute to attention-deficient hyperactivity disorder symptoms [31]. In addition to working memory and attention function, the MCC is also involved in executive function. In an rs-fMRI study, it was demonstrated that the MCC was a core region of the executive function network that mediated the processing of episodic memory [27]. Recently, an increasing number of clinical studies have reported that patients with abnormal brain function in the MCC were more vulnerable to cognitive impairment. Li et al. [32] published an important study in which patients with Parkinson's disease and mild cognitive impairment, compared to healthy subjects, presented increased spontaneous synchrony in the bilateral MCC. In an fMRI study evaluating functional compensation throughout the progression of Alzheimer's disease, it was found that the MCC had stronger FC with several brain regions in the asymptomatic preclinical group and mild cognitive impairment group. The results shed light on the compensatory role of the MCC across the pathophysiological continuum of Alzheimer's disease [33]. Taken together, these findings indicated that the MCC plays crucial roles in the pathogenesis of various diseases associated with cognitive dysfunction.

In addition to the ALFF findings, whole-brain seed-based FC analysis showed that the patients with lower preoperative FC between the bilateral MCC and left calcarine were more prone to cognitive impairment following surgery. The calcarine, as part of the primary visual cortex, includes the primary visual pathway contributing to visual-spatial processing, perception, episodic memory retrieval, and attention [34–36]. In addition, it has been reported that the primary visual cortex is vital for maintaining information about a stimulus in working memory [37–39]. Using fMRI, Harrison and Tong [40] demonstrated that primary visual areas sustained information for periods of many seconds, indicating that their function was not restricted to sensory processing but extended to the maintenance of visual features and patterns in memory. A large body of studies has suggested that abnormal alterations in regional activity and functional connectivity of the calcarine exist in patients with cognitive impairment [41, 42]. Moon and Jeong [42] confirmed that lower spontaneous activities in the calcarine were related to cognitive impairment. In addition, it was demonstrated that patients with both amnestic cognitive impairment and major depression disorder displayed abnormal effective connectivity from the right amygdala to the calcarine gyrus [35]. Notably, MCC-calcarine functional connections have been reported in previous rs-fMRI studies. Zuo et al. [43] demonstrated that agenesis of the corpus callosum patients with severe cognitive deficits exhibited lower FC between the MCC and calcarine. In the present study, a battery of neuropsychological tests was used to assess patients' cognitive function. The DSST mainly examines working memory, visual-motor coordination, and visual-spatial scanning [44]. The TMT-A is designed to assess attention and visual search speed abilities [45]. We found that the DNR patients exhibited lower DSST and TMT-A scores following surgery than the non-DNR patients. The results indicated that older patients with abnormal changes in synchronous spontaneous fluctuations between the bilateral MCC and left calcarine were more susceptible to deterioration in visual cognitive function following major noncardiac surgery.

A previous study demonstrated that a higher education level was associated with a lower incidence of DNR [46]. Education has been the most commonly considered cognitive reserve indicator. Patients with a higher cognitive reserve may be able to better cope with neuropathological insult due to aging and disease [47]. Patients with lower cognitive reserve may have greater subclinical neuropathology, which could be exacerbated by surgery and become expressed as cognitive deficits [48]. Consistent with previous studies, the present study also found that a longer education duration decreased the risk of DNR following surgery.

Recent studies have examined the preoperative alterations in the brain structure in patients with postoperative cognitive decline. However, the results have been inconsistent. Maekawa et al. [11] enrolled 28 older patients undergoing cardiac surgery and found reduced preoperative gray matter in the hippocampus and entorhinal cortex in the patients who developed postoperative cognitive dysfunction. In contrast, other studies did not find any abnormalities in gray matter in patients who developed postoperative cognitive disorders [49, 50]. A structural MRI study reported no statistically significant differences in hippocampal or whole-brain volume between patients with and without delirium [50]. In the present study, there were no brain areas showing significant differences in gray matter volume between the DNR patients and non-DNR patients.

There were several limitations in the present study. First, MRI was not performed after surgery because most patients refused to receive another MRI scan after surgery. Therefore, we were unable to investigate whether spontaneous brain activity changed following surgery. Second, postoperative neuropsychological tests were performed at 7-14 days after surgery. This timeframe was too early to make a diagnosis of postoperative cognitive impairment, since many influencing factors, such as pain, inflammatory activity, and postoperative delirium, may still exist at that time. In the present study, the patients did not have clinically significant pain or delirium when they returned to the hospital to receive neuropsychological testing. However, we could not completely rule out the effects of these influencing factors. Third, the MCC has been associated with depression, and depression has been associated with poor cognitive function. Depression was not assessed in the present study, although patients with diagnosed psychiatric disorders and taking antidepressants were excluded. Therefore, the results should be interpreted in the context of these caveats.

## 5. Conclusion

The present study demonstrated that preoperative changes in spontaneous brain activity and FC were detected in older patients who developed DNR after noncardiac surgery. The present fMRI study identified possible preoperative neuroimaging risk factors for DNR and provided impetus for further research seeking effective tools to screen patients at high risk for DNR.

## Figures and Tables

**Table 1:** Baseline characteristics.

	DNR (*n* = 16)	Non-DNR (*n* = 58)	*P*
Characteristics			
Age (years)	63.5 (62.0, 67.0)	64.0 (61.0, 68.3)	0.598
Sex (male/female)	12/4	29/29	0.075
Education (year)	6 (6, 9)	9 (9, 12)	0.002
Height (m)	1.68 (0.08)	1.65 (0.08)	0.185
Weight (kg)	59.0 (50.0, 70.5)	60.0 (54.8, 70.0)	0.324
BMI ≥ 24, *n*	3 (18.8)	19 (32.8)	0.437
Smoking, *n*	7 (43.8)	15 (25.9)	0.281
Surgical history, *n*	6 (37.5)	28 (48.3)	0.444
Comorbidities, *n*			
Hypertension	7 (43.8)	24 (41.4)	0.865
Diabetes mellitus	1 (6.3)	7 (12.1)	0.835
Hepatorenal dysfunction	4 (25.0)	10 (17.2)	0.733
Anemia	5 (31.3)	10 (17.2)	0.309
COPD	5 (31.3)	14 (24.1)	0.800
Peptic ulcer disease	4 (25.0)	5 (8.6)	0.179
Intraoperative conditions			
Surgical duration (min)	120 (114, 166)	95 (70, 135)	0.055
Nature of surgery (minimally invasive surgery/open surgery)	12/4	54/4	0.107
Propofol (mg)	300 (53, 844)	324 (68, 678)	0.974
Sufentanil (*μ*g)	35 (21, 49)	30 (25, 35)	0.148
Remifentanil (*μ*g)	1506 (1253, 2113)	1195 (738, 1800)	0.059

Continuous variables are presented as mean (standard deviation) or median (interquartile range). BMI: body mass index; COPD: chronic obstructive pulmonary disease; DNR: delayed neurocognitive recovery.

**Table 2:** Neuropsychological results.

	DNR (*n* = 16)	Non-DNR (*n* = 58)	*P*
MMSE			
Baseline	27 (25, 28)	27 (26, 29)	0.579
Follow-up	26.0 (23.3, 27.8)	28.0 (26.0, 28.3)	0.032
DSF			
Baseline	7.5 (6.0, 9.0)	8.0 (7.0, 9.0)	0.534
Follow-up	6.5 (5.0, 8.0)	8.0 (7.0, 8.0)	0.008
DSB			
Baseline	4.0 (3.0, 4.8)	4.0 (3.0, 4.0)	0.563
Follow-up	3 (3, 4)	4 (3, 4)	<0.001
DSST			
Baseline	21.5 (16.0, 31.0)	28.5 (21.8, 35.0)	0.038
Follow-up	19.5 (15.0, 24.0)	30.0 (24.8, 35.0)	<0.001
VFT			
Baseline	14.9 (4.2)	15.8 (3.0)	0.472
Follow-up	12.5 (10.0, 14.0)	15.0 (13.8, 19.0)	0.002
TMT-A (seconds)			
Baseline	48.5 (37.8, 76.5)	44.5 (34.8, 60.5)	0.245
Follow-up	76.0 (48.5, 104.5)	42.0 (33.0, 54.0)	<0.001

Continuous variables are presented as mean (standard deviation) or median (interquartile range). DNR: delayed neurocognitive recovery; DSB: Digit Span Backwards; DSF: Digit Span Forwards; DSST: Digit Symbol Substitution Test; MMSE: Mini-Mental State Examination; TMT-A: Trail Making Test-part A; VFT: Verbal Fluency Test.

**Table 3:** Risk factors for DNR.

Reference variables	Univariate analysis	Multivariate analysis	
	*P*	OR (95% CI)	*P*
Education duration	0.005	0.57 (0.41-0.80)	0.001
Sex	0.075	—	0.054
ALFF in bilateral MCC	0.004	3.11 (1.30-7.45)	0.011
ALFF in left FFG	0.008	—	0.091
Age	0.661	—	0.077
FC between MCC and left calcarine	0.008	0.40 (0.18-0.92)	0.031

ALFF: amplitude of low-frequency fluctuations; CI: confidence interval; DNR: delayed neurocognitive recovery; FC: functional connectivity; FFG: fusiform gyrus; MCC: middle cingulate cortex; OR: odds ratio.

## Data Availability

The data that support the findings of this study are available upon reasonable request by contact with the corresponding author, Weidong Gu.

## Supplementary Materials

Supplementary Materials Supplemental Table 1: brain regions exhibiting higher ALFF and lower MCC-seeded FC prior to surgery in the DNR patients compared to those in the non-DNR patients. Supplemental Table 2: partial correlations between fMRI indices and baseline cognitive data.
