# Supplementary material for: Preoperative Altered Spontaneous Brain Activity and Functional Connectivity Were Independent Risk Factors for Delayed Neurocognitive Recovery in Older Adults Undergoing Noncardiac Surgery
Source: Neural Plast. 2020 Jun 16;2020:9796419. doi: 10.1155/2020/9796419 (PMC7315267; doi:10.1155/2020/9796419)
Supplement: Supplementary Materials — Supplemental Table 1: brain regions exhibiting higher ALFF and lower MCC-seeded FC prior to surgery in the DNR patients compared to those in the non-DNR patients. Supplemental Table 2: partial correlations between fMRI indices and baseline cognitive data. [file 9796419.f1.docx]

**Supplemental materials**

Supplemental Table 1. Brain regions exhibiting higher ALFF and lower MCC-seeded FC prior to surgery in the DNR patients compared to that in the non-DNR patients.

| Brain region | BA | Hem | MNI peak point coordinates | | | T-value | cluster size |
| --- | --- | --- | --- | --- | --- | --- | --- |
|  |  |  | X | Y | Z |  |  |
| **ALFF analysis** | | | | | | | |
| MCC | 24 | L&R | 0 | -33 | 39 | 5.049 | 56 |
| FFG | 37 | L | -42 | -57 | -12 | 4.612 | 49 |
| **MCC-seeded FC analysis** | | | | | | | |
| calcarine | 17 | L | -9 | -87 | -6 | -4.388 | 52 |

ALFF = amplitude of low-frequency fluctuations; BA = brodmann area; DNR = delayed neurocognitive recovery; FC = functional connectivity; FFG = fusiform gyrus; Hem = hemisphere; L = left; MCC = middle cingulate cortex; MNI = Montreal Neurological Institute; R = right.

Supplemental Table 2. Partial correlations between fMRI indices and baseline cognitive data

| Baseline scores | ALFF values | | | | FC values between MCC and calcarine | |
| --- | --- | --- | --- | --- | --- | --- |
|  | MCC | | FFG | |  |  |
|  | r | *P* | r | *P* | r | *P* |
| MMSE | -0.207 | 0.084 | -0.036 | 0.768 | 0.038 | 0.752 |
| DSF | 0.010 | 0.933 | -0.187 | 0.119 | 0.044 | 0.716 |
| DSB | -0.018 | 0.880 | -0.066 | 0.583 | -0.063 | 0.604 |
| DSST | -0.025 | 0.835 | -0.025 | 0.836 | -0.034 | 0.781 |
| TMT-A | -0.074 | 0.539 | 0.095 | 0.429 | 0.05 | 0.680 |
| VFT | 0.030 | 0.803 | 0.161 | 0.181 | -0.106 | 0.381 |

ALFF = amplitude of low-frequency fluctuations; DSB = Digit Span Backwards; DSF = Digit Span Forwards; DSST = Digit Symbol Substitution Test; FC = functional connectivity; FFG = fusiform gyrus; MCC = middle cingulate cortex; MMSE = Mini-Mental State Examination; TMT-A = Trail Making Test part A; VFT = Verbal Fluency Test.
